# The impact of online teaching in behavior intention for college students in Taiwan

**DOI:** 10.3389/fpsyg.2022.911262

**Published:** 2022-08-26

**Authors:** Chih-Hung Tseng, Chin-Hsien Hsu, Jing-Wei Liu, Ching-Tang Wang

**Affiliations:** ^1^Department of Leisure Industry Management, National Chin-Yi University of Technology, Taichung, Taiwan; ^2^Department of Industrial Engineering and Management, National Taipei University of Technology, Taipei, Taiwan; ^3^Department of Sport Information and Communication, National Taiwan University of Sport, Taichung, Taiwan

**Keywords:** online teaching, trust, convenience, perceived critical mass, technology acceptance model

## Abstract

This paper aims to explore the change of learning mode of college students from physical courses to online courses due to the impact of the COVID-19 pandemic. The questionnaire survey method is used to conduct research on the behavior intentions of college students in online teaching under the pandemic. A total of 600 questionnaires are distributed, and 530 questionnaires are collected, for a recovery rate of 88%. A total of 493 supported questionnaires are received, for an effective recovery rate of 93%. Descriptive statistics of data analysis are used to analyze the distribution of background variables of college students, and a structural equation model is used to analyze and verify the impact of external variables (trust, convenience, perceived critical mass) on the technology acceptance model (perceived usefulness, perceived ease of use, attitude, and behavior intention). The results found no significant impact of the trust of college students in online teaching on the perceived usefulness, and significant impact of trust on the perceived ease of use. There is significant impact of convenience on perceived usefulness, and no significant impact of convenience on perceived ease of use. There is no significant impact of perceived critical mass on perceived usefulness, and significant impact of perceived critical mass on perceived ease of use. There is significant impact of perceived ease of use on perceived usefulness, and significant impact of perceived usefulness on attitude. There is significant impact of perceived ease of use on attitude, and significant impact of attitude on behavior intention. Based on the research results, practical suggestions and research suggestions are proposed in this research, which can be used as a reference for college students to use online courses for learning.

## Introduction

In December 2019, a number of viral pneumonia cases of unknown origin were identified in Wuhan, Hubei Province, followed by imported cases from Wuhan into Thailand, Japan, and South Korea. The pandemic then spread rapidly to the whole world. The first imported confirmed case from abroad was discovered in Taiwan on January 21, 2020. As the pandemic intensified, all countries took strict measures to prevent the spread of COVID-19. Nevertheless, the COVID-19 pandemic spread so fast that it had a huge impact on education, the economy, society, and entertainment world wide. Later, after successive infection cases of unknown origin and cluster events occurred in Taipei City and New Taipei City, the Central Epidemic Command Center began to increase the epidemic alert to Level III on May 15, 2021 after analysis showed that the cases of infection in communities would spread broadly. Various restrictions were employed, such as the suspension of opening elementary and junior high schools nationwide to the outside world and the suspension of activities of associations nationwide (Centers for Disease Control, Ministry of Health and Welfare, 2021). As educational sites, schools require a more active strategy in the face of unexpected public health events. Therefore, the Ministry of Education (2021) instructed that, in response to the escalation of Taiwan’s pandemic alert, in order to reduce the risk of cluster infections and safeguard the health and learning rights of teachers and students, it issued an order that all levels of schools across the country, as well as public and private kindergartens, should stop giving offline lessons from May 19, 2021 to May 28, 2021, colleges and schools below high schools should adopt online teaching, and students should study at home, thus, online courses became the official courses. In fact, remote teaching had been in practice for a long time before the outbreak (Lo and Chen, 2021), which is different from physical teaching and allows teachers and students to have the opportunity to teach and learn through different online teaching platforms. Radha et al. (2020) also pointed out that digital learning in the post-pandemic era could provide a more flexible learning space for each learner and teacher; however, there are some problems with the implementation of online teaching. Chang and Lee (2021) pointed out that there are several problems for students during online teaching: (1) When students have some problems in online learning, they may not be able to immediately obtain solutions. (2) When students synchronize online courses, they may miss class time. (3) Students may be easily distracted in non-school settings. (4) The infrastructure of online learning may be insufficient. Among the above problems, students’ learning attitudes and the attributes of online teaching platforms play an important role in students’ learning effect. In addition, students’ digital divide and the lack of hardware equipment may lead to unsatisfactory learning effects; for example, some students are not familiar with the platform operation interface and may lack the basic ability of computer operation; the learning process is interrupted by the lack of internet access, and the lack of computers or tablet devices is still to be overcome. Thus, exploring students’ trust in online teaching platforms and their perceptions of the convenience of online teaching platforms are the first motivations of this research. In addition, in an era of social media and the Internet, students’ experience of using online teaching platforms is often constructed through peers or other significant people, thus, exploring how the critical mass provides the necessary conditions for the use of online teaching platforms is the second motivation of this research. In addition, in order to further construct the model of students adopting the online teaching platform, as based on the technology acceptance model proposed by Davis (1986), this paper further explored the impacts of trust, convenience, and perceived critical mass of the online teaching platform on perceived ease of use and perceived usefulness, with students as the research subject.

Moreover, students’ digital divide and the differences in hardware equipment may also cause the learning effect to be less than expected. For example, if the internet connection is not smooth during online teaching, the operation interface of the online teaching platform will be difficult to use. Whether these problems generated in the online teaching process affect students’ attitude regarding the online teaching system is worthy of further discussion. Therefore, the third research motivation of this paper is to explore the impacts of perceived usefulness and perceived ease of use on students’ attitudes toward online teaching systems.

It can be seen from relevant literature that the technology acceptance model is often used to explore the use of new technologies (Lin and Chen, 2021; Tsai and Wang, 2021; Vahdat et al., 2021), thus, this paper also explores the relevant topics of college students’ adoption of online teaching by adopting the technology acceptance model. In addition, more research has recently been conducted on topics related to online teaching in the post-pandemic period. For example, Han (2021) explored the learning adaptation of online teaching and learning motivation of higher elementary school students under the COVID-19 pandemic, with students as the research subject; Liu (2020) explored the teaching practices in response to the requirements of online teaching, as issued by the US government, as well as the responding strategies of schools from mid-March 2020, and explored students’ learning motivation with students from the District of Columbia International School, DCI Middle School as the research subject. Based on the Push-Pull-Mooring theory, Chen (2019a) explored whether online teaching in universities can replace traditional offline teaching under the pandemic through questionnaire surveys completed by college students. It can be seen from the above relevant literature that under the current pandemic situation, while there are many research results regarding online teaching related topics, there is little related research on college students’ adoption of online teaching platforms. Therefore, the fourth motivation of this paper is to explore the impact of college students’ attitudes toward online teaching systems on behavior intention.

## Literature review

### Trust

Consumers’ consumption of a certain product or acceptance of a certain service is often based on their trust in merchants or enterprises. Especially in contemporary society, both the complex information of goods and services and information asymmetry make consumers focus more on their purchasing decisions. Therefore, trust is often the basis for a transaction between a buyer and a seller. Doney and Cannon (1997) held a similar view, and pointed out that trust comes from reliability and goodwill, as perceived by the subject. Abbas et al. (2018) further pointed out that the trust factor plays a significant role in a relationship, and when users trust technology, they will intensify their adoption behavior, thus, it can be seen that trust plays a significant role in consumers’ minds. McKnight et al. (2002) pointed out that the lack of trust makes many consumers hesitate to express the behaviors necessary for the widespread use of mobile payments, such as sharing personal information and using mobile payment. In other words, when consumers, stores, and enterprises establish trust, they may establish a good relationship with each other. Reichheld and Schefter (2000) echoed this view and pointed out that when a website is trusted, most consumers are willing to have the consumer behavior on the website. Kim et al. (2009) established an Initial Trust Model (ITM) and formulated four key antecedents of initial trust, namely Relative Benefits (RB), Structural Assurances, (SA), Propensity to Trust (PT), and Firm Reputation (FR). It can be seen that the process of consumer trust involves many aspects. Menon et al. (2000) held the same view, and suggested that the tendency to trust will vary according to different growth environments, personal characteristics, and cultural backgrounds, just as people who establish personal dependence on others will have the psychological effect of believing that others are reliable. The higher the tendency to trust others, the deeper the trust will be in receiving information from others. Scholars have different views on the measurement of trust. For example, Singh and Sirdeshmukh (2000) mentioned that the establishment of consumer trust requires competence and benevolence, while Michell et al. (1998) pointed out that trust can be measured by affective and cognitive dimensions, of which, the affective dimension is integrity and fairness, and the cognitive dimension is reliability and satisfaction.

### Convenience

The concept of convenience can be used as an entry point to determine how much time and effort consumers spend in obtaining a product or using a service. Butcher et al. (2002) pointed out that convenience is a conversion relationship between acquisition and effort (including space and time), such as the convenience of store location and effective service will affect consumers’ purchase intentions. Brown (1989) held a similar view, and proposed that convenience does not just mean saving time, and suggested that the more convenience a product or service provider can offer, the more attention consumers will pay to the products and services and the more willing they will be to buy and use. He held that convenience should include five major dimensions: (1) Time convenience, which means that companies provide products to consumers at the most convenient time; (2) Location convenience, which means that companies provide products to consumers in more convenient places; (3) Acquisition convenience, which means that companies provide convenient product purchase channels to consumers; (4) Use convenience, which means that consumers feel the companies’ products are convenient to use; (5) Execution convenience, which means that companies’ products provide the convenience of customers choosing to execute by themselves or by others. Berry et al. (2002) advocated that convenience should include five dimensions based on the various stages of activities that consumers experience when buying or using services: (1) Decision convenience, which refers to the time and effort consumers spend deciding how to get the service they want; (2) Acquisition convenience, which refers to the paid time and effort perceived by consumers when using services provided by companies; (3) Transaction convenience, which refers to the paid time and effort perceived by consumers when conducting transactions; (4) Benefit convenience, which refers to the paid time and effort perceived by consumers when experiencing the core benefits of the services provided by companies; (5) Follow-up benefit convenience, which refers to the paid time and effort perceived by consumers when contacting the companies again after using their services. It can be seen from the above classifications of convenience that the time and effort spent by consumers in obtaining products or services are the main focuses of the requirements in the concept of convenience, as well as the discussion basis in the relevant research of service segmentation (Keh and Pang, 2010). Although different scholars have different classifications of convenience, Hornik (1984) pointed out that there are multiple convenience attributes that simultaneously occur in the products and services of convenience, and consumers can decide how much time or effort they want to spend in obtaining this product or service when purchasing, such as an ATM that offers the convenience of location, acquisition, and use of various attributes, in addition to the convenience of time.

### Perceived critical mass

With the prosperity of We Media, the flow of goods and services has largely broken the restrictions of time, space, and territory, especially at a time when the interactivity of new media has become a phenomenon that cannot be ignored. In 1983, Oliver proposed the concept of critical mass and held that critical mass means that people would have a certain social tendency after the number of participants exceeds a certain threshold. Shapiro and Varian (1988) described consumers’ usage behavior of information products with an S-curve, which was divided into an initial flat period, a steep rise period when the positive feedback cycle starts, and a recovery flat period after saturation. There is also a turning point in the curve that represents the user’s key quantity, and beyond this point, a positive cycle starts and the use value increases rapidly. However, as the critical mass of the actual system is difficult to define, Markus (1992) further put forward a view on critical mass, and proposed the concept of perceived critical mass after researching interactive media. He held that when the number of participants in the system reaches the majority in a user’s cognition, the user will have a tendency to use the system. Hsu (2021) also pointed out that perceived critical mass (PCM) is used to perceive the degree of adoption of new technologies by the public, which can be used as a reference to evaluate whether an individual adopts a new technology, and has an important impact on users’ decision of adopting a new technology. Relevant researches include communication software, social media, mobile devices, and online games. However, the impacts of perceived critical mass on the use of the Internet of Things technology are yet to be explored. Taking this paper as an example, as online teaching has become the main teaching method under the pandemic, both teachers and students will influence each other in the process of using online teaching. When most groups think that an online teaching platform is easy to use, relevant personnel in other educational fields will be more willing to adopt the platform. This phenomenon also echoes the view of Lou et al. (2000), who advocated from a sociological point of view that when individuals form a perceived critical mass, there will be a tendency to use the system due to the influence of group information and norms. He also pointed out that an individual’s use of communication technology is influenced by the number of users according to their perception, and not the actual number of users. It can be seen from the above literature that people are often influenced by groups and society to use new technology, and they will naturally pay attention to users’ subjective feelings about the new technology. When others are positive about a technology after use, the technology is more likely to become the mainstream technology of the group due to users’ recognition.

### Technology acceptance model

Theories that explore people’s usage behavior are constantly developing with the times. From the theory of reasoned action (TRA) proposed by Fishbein and Ajzen (1975), and the theory of planned behavior (TPB) proposed by Ajzen (1991), to the technology acceptance model (TAM) proposed by Davis (1989), which all demonstrate that the public’s usage behavior of new technology has always been a topic of concern for experts and scholars. Based on the theory of reasoned action, as proposed by Fishbein and Ajzen (1975) and Davis et al. (1989) put forward the technology acceptance model (TAM), which is based on a stable set of beliefs and can generally extrapolate to different computer systems and users, thereby removing the need to generate new beliefs each time. Morris and Dillon (1997) also held the same view and pointed out that regardless of whether the system is practical, the technology acceptance model can provide researchers with a simple, more cost-effective, and time-saving method to estimate the degree of success of the system, and researchers can evaluate whether or not the system is good. The technology acceptance model includes five main dimensions, perceived usefulness, perceived ease of use, attitude, behavioral intention, and usage behaviors: (1) Perceived usefulness: Users subjectively hold that the use of a specific information system can improve their current or future work performance. (2) Perceived ease of use: Users subjectively hold that fewer efforts are made when using a specific information system. (3) Attitude: Distinguishable characteristics in which an object, person, event, institution, or other person is treated pleasantly or unpleasantly (positively or negatively). (4) Behavioral intention: Measure the intensity of willingness of a specific behavior during use. (5) Usage behavior: For actual use (Davis, 1989). Specifically, perceived usefulness will be affected by the perceived ease of use, and perceived ease of use has significant and positive impact on perceived usefulness. In other words, people will tend to use tools that they think can help them do their jobs better and do more work with the same effort to achieve target performance quickly, thus, it is believed that there is an influential relationship between the two. As the technology acceptance model becomes more widely used, its shortcomings have been identified by relevant research materials. Venkatesh and Davis (2000) extended the original technology acceptance model and proposed a second-generation technology acceptance model with seven dimensions, as follows: (1) Subjective norm: users will be influenced by people important to them (elders, teachers, relatives, and friends) when considering engaging in a certain behavior; (2) Experience: the experience gained from past experiences; (3) Voluntary: users follow their hearts to experience a new system or new thing without compulsion; (4) Image: Perceiving that the use of innovative systems can improve social status; (5) Work relevance: considering the degree to which new technology can be applied to their work; (6) Output quality: perceiving that the system can help achieve better work output; (7) Results display: perceiving that the use of innovation system results can be easily observed and the degree of clarity. According to the definitions of the above dimensions, the second-generation technology acceptance model makes up for the shortcomings of the first-generation technology acceptance model, and provides a more complete application to subsequent relevant research.

### Relationship between variables

In the model constructed in this research, the relationship between variables is supported by previous relevant researches: There is a positive impact of trust on perceived usefulness and perceived ease of use (Harrigan et al., 2021; Sari et al., 2021); there is a positive impact of convenience on perceived usefulness (Yoon and Kim, 2007; Chang et al., 2011; Hsu, 2017; Shen et al., 2021); there is a positive impact of convenience on perceived ease of use (Hsu, 2017; Shen et al., 2021); there is a positive impact of perceived critical mass on perceived usefulness (Lee et al., 2013; Shyh, 2015; Hsu, 2020); there is a positive impact of perceived critical mass on perceived ease of use (Lee et al., 2013; Shyh, 2015); there is a significantly positive impact of perceived ease of use on perceived usefulness (Byun et al., 2018); there are positive impacts of perceived ease of use and perceived usefulness on attitude (Vijayasarathy, 2004; Chen et al., 2014; Chen, 2019b; Islami et al., 2021); there is a positive impact of attitude on behavior intention (Chang, 2021; Yang, 2021).

### Definition

Trust is the user’s belief that the system is reliable and upright in the process of using the network system; Convenience is the degree to which users feel that the operation, method, time and other processes are convenient when using online teaching; Perceived critical mass (PCM) is used to perceive the degree to which new technologies are adopted by the general public; The Technology Acceptance Model (TAM) is used in conjunction with the application context of the new technology information system, and is suitable for explaining and predicting the behavioral intention pattern of users’ acceptance of the new information technology system.

## Research method

### Research structure

**Figure 1:**
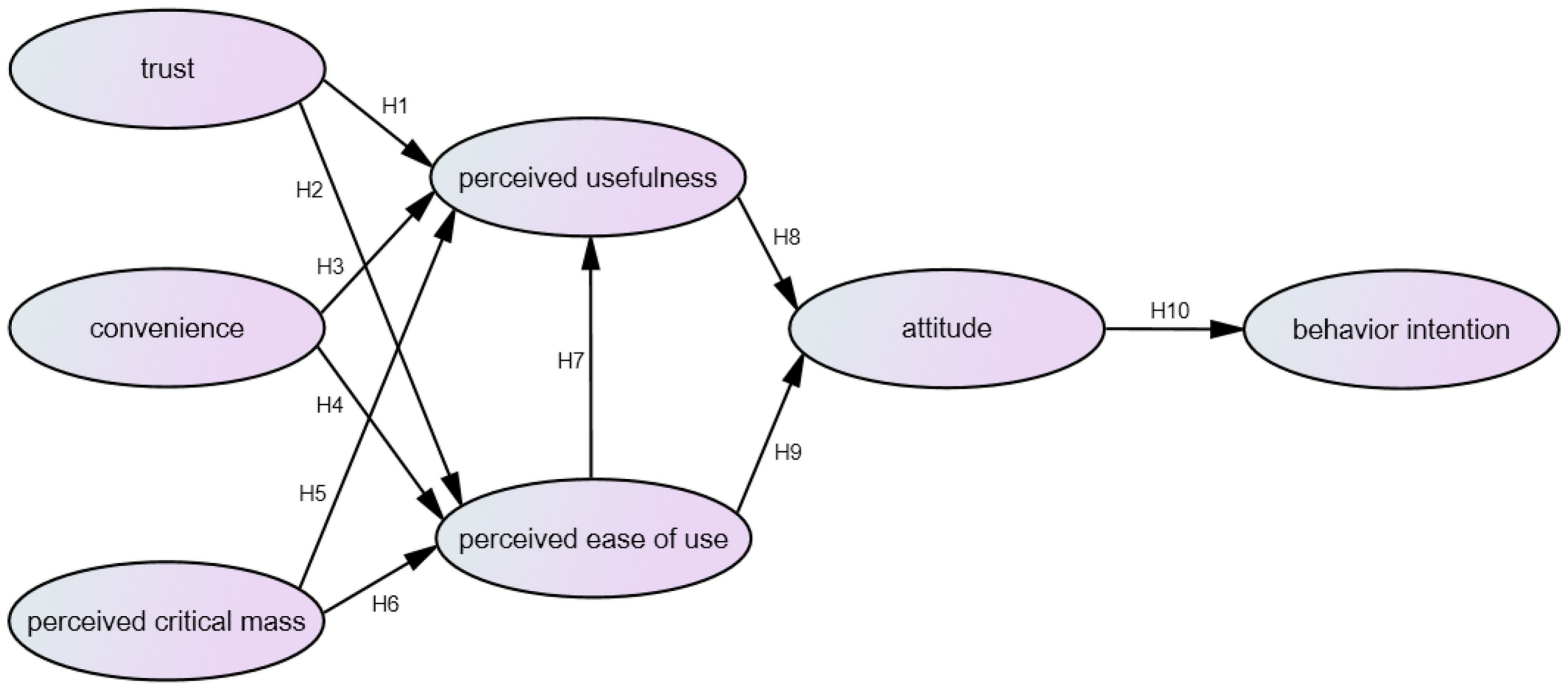
Research structure.

This study the impact of online teaching in behavior intention for college students. According to the research purpose and related literature, the research structure proposed is shown in Figure 1.

### The following 10 research hypotheses were put forward by this study based on the research purposes and related literature

H1: trust has a significant impact on perceived usefulness.

H2: trust has a significant impact on perceived ease of use.

H3: convenience has a significant impact on perceived usefulness.

H4: convenience has a significant impact on perceived ease of use.

H5: perceived critical mass has a significant impact on perceived usefulness.

H6: perceived critical mass has a significant impact on perceived ease of use.

H7: perceived ease of use has a significant impact on perceived usefulness.

H8: perceived usefulness has a significant impact on attitude.

H9: perceived ease of use has a significant impact on attitude.

H10: attitude has a significant impact on behavior intention.

### Research subjects

This study was investigate the behavior intention of college students regarding online teaching under the influence of the COVID-19 pandemic. The research subjects were college and university students. The survey was conducted with questionnaire. A total of 600 questionnaires were distributed, and 530 questionnaires were collected with a recovery rate of 88%. A total of 493 supported questionnaires were received with an effective recovery rate of 93%.

### Research tools

The content of this research questionnaire were revised and complied mainly based on the relevant literature of questionnaires by Gefen et al. (2003), Venkatesh et al. (2003), Lou et al. (2005), Van Slyke et al. (2007), Wu et al. (2008), Rauniar et al. (2014), Kang (2019), Ou (2019), Hsu (2021), Liao (2021), and Shen (2021). There were five sections with a total of 42 items in the questionnaire. The Likert five-point scale was adopted. Each item was given a rate of 1 to 5 from “Strongly disagree” to “Strongly agree.”

### Data processing and analysis

After the questionnaires being collected, invalid questionnaires were eliminated according to the criteria of valid questionnaires. SPSS 22.0 statistical software was used to establish the data file. Then, AMOS 22.0 statistical software was used to analyze the correlations between variables.

## Research results

### Sample characteristics

This study took students from colleges and universities as the test samples. There were a total of 493 valid samples. Among them, in terms of gender, there were 297 females, accounting for 60.2% of the valid samples, and 196 Male, accounting for 39.8% of the valid samples. In terms of grade, most of them (106) were in junior, accounting for 21.5% of the valid samples, and only others 15 people were in others, accounting for 3.0% of the valid samples. Regarding school, 294 people were in the public university, accounting for 59.6% of the valid samples, and the least number of subjects (37) was in the private university, accounting for 7.5% of the valid samples. In terms of Location of schools, there was a total of 256 people were in the central region, accounting for 51.9% of the valid samples, and only 14 people with South, accounting for 2.8% of the valid samples. In terms of the most frequently used video software, Google Meet had a maximum of 400 users, which accounted for 81.1% of the valid samples, and only 14 people with others, accounting for 2.8% of the valid samples. The details are shown in Table 1.

**Table 1:** Subjects’ characteristics.

Variable	Category	Number of times	Percentage %	Cumulative percentage %
Gender	Male	196	39.8	39.8
	Female	297	60.2	100.0
Grade	Freshman	75	15.2	15.2
	Sophomore	96	19.5	34.7
	Junior	106	21.5	56.2
	Senior	76	15.4	71.6
	Graduate school	92	18.7	90.3
	PhD	33	6.7	97.0
	Others	15	3.0	100.0
School	Public university	294	59.6	59.6
	National university of science and technology	80	16.2	75.9
	Private university	37	7.5	83.4
	Private university of science and technology	82	16.6	100.0
Location of schools	North	84	17.0	17.0
	Central	256	51.9	69.0
	South	14	2.8	71.8
	East	95	19.3	91.1
	Outlying islands	44	8.9	100.0
The most frequently used video software	Microsoft Teams	47	9.5	9.5
	Google Meet	400	81.1	90.7
	Line	32	6.5	97.2
	Others	14	2.8	100.0

### Measurement model analysis

CFA was adopted to examine the reliability and validity of the questionnaire. Modifications were made based on the Modification Indices (MI; Chen, 2007). B4 of the Trust Scale, C3 of the convenience Scale, D4 and D5 of the perceived critical mass, as well as A7, A10, A13, and A14 of the Technology Acceptance Model Scale were, hence, deleted in this study.

#### Test of convergent validity

Fornell and Larcker (1981) and Bagozzi and Yi (1988) that the composite reliability (CR) and average variance extracted (AVE) of the questionnaire dimensions shall be measured in the convergent validity test. For good convergent validity of a questionnaire, it is recommended that the CR value should be greater than 0.6 and the AVE value should be greater than 0.5. In this study, the convergent validity test was conducted for the Trust, the convenience, and the perceived critical mass and Technology Acceptance Model. The factor loadings of all the dimensions were between 0.67 and 0.95, the CR values were between 0.80 and 0.93, and the AVE values were between 0.58 and 0.78, conforming to the convergent validity standard recommended (Fornell and Larcker, 1981; Bagozzi and Yi, 1988; Hair et al., 1998). This means the convergent validity of the questionnaire in this study is good, as shown in Tables 2–5.

**Table 2:** Technology acceptance model—confirmatory factor analysis.

Dimension	Indicator	Standardized load	Non-standardized load	S.E.	C.R. (*t*-value)	*P*	SMC	C.R.	AVE
Perceived ease of use	A1	0.83	1.00				0.69	0.93	0.78
	A2	0.91	1.18	0.05	26.15	***	0.83		
	A3	0.92	1.19	0.05	26.45	***	0.84		
	A4	0.89	1.22	0.05	25.32	***	0.79		
Perceived usefulness	A5	0.79	1.00				0.63	0.87	0.69
	A6	0.87	1.00	0.05	21.77	***	0.75		
	A8	0.84	0.97	0.05	20.91	***	0.71		
Attitude of use	A9	0.77	1.00				0.59	0.90	0.76
	A11	0.94	1.14	0.05	22.78	***	0.89		
	A12	0.91	1.11	0.05	21.98	***	0.82		
Behavior intention	A15	0.84	1.00				0.71	0.90	0.75
	A16	0.87	1.03	0.04	24.44	***	0.76		
	A17	0.89	0.99	0.04	24.77	***	0.79		

***p < 0.001.

**Table 3:** Trust confirmatory factor analysis.

Dimension	Indicator	Standardized load	Non-standardized load	S.E.	C.R. (*t*-value)	*P*	SMC	C.R.	AVE
Trust	B1	0.78	1.00				0.62	0.92	0.75
	B2	0.93	1.06	0.04	23.84	***	0.86		
	B3	0.92	1.02	0.04	23.56	***	0.85		
	B5	0.84	0.94	0.04	20.97	***	0.71		

***p < 0.001.

**Table 4:** Convenience confirmatory factor analysis.

Dimension	Indicator	Standardized load	Non-standardized load	S.E.	C.R. (*t*-value)	*P*	SMC	C.R.	AVE
Convenience	C1	0.72	1.00				0.52	0.80	0.58
	C2	0.88	1.41	0.10	13.52	***	0.77		
	C4	0.67	1.22	0.09	13.31	***	0.45		

***p < 0.001.

**Table 5:** Perceived critical mass confirmatory factor analysis.

Dimension	Indicator	Standardized load	Non-standardized load	S.E.	C.R. (*t*-value)	*P*	SMC	C.R.	AVE
perceived critical mass	D1	0.88	1.00				0.77	0.93	0.77
	D2	0.91	1.01	0.03	29.82	***	0.83		
	D3	0.95	1.06	0.03	31.50	***	0.89		
	D6	0.77	0.86	0.04	21.34	***	0.59		

***p < 0.001.

#### Discriminant validity

The discriminant validity of the study was tested with the confidence interval method (Bootstrap). The questionnaire dimensions were correlated depends on the value of the Pearson correlation coefficient confidence interval among the questionnaire dimensions of this study. The value should not contain 1. The results showed that the confidence interval between the questionnaire dimensions was missing the value of 1, indicating that the questionnaire dimensions of this study have significant discriminant validity (Torkzadeh et al., 2003), as shown in Table 6.

**Table 6:** Technology acceptance model—bootstrap correlation coefficient 95% confidence interval.

Parameters			Estimate	Bias-corrected		Percentile method	
				Lower bound	Upper bound	Lower bound	Upper bound
Perceived ease of use	↔	Perceived usefulness	0.91	0.88	0.94	0.88	0.94
Perceived ease of use	↔	Attitude of use	0.42	0.34	0.50	0.34	0.50
Perceived ease of use	↔	Behavior intention	0.80	0.75	0.85	0.75	0.85
Perceived usefulness	↔	Attitude of use	0.56	0.47	0.65	0.47	0.64
Perceived usefulness	↔	Behavior intention	0.89	0.84	0.93	0.84	0.92
Attitude of use	↔	Behavior intention	0.64	0.57	0.71	0.56	0.71

#### Structural equation model analysis

The structural model analysis of Hair et al. (1998) was used to examine the overall model fit. The overall model fit was tested with the seven indicators of chi-square value (χ2), the ratio of χ2 to its degrees of freedom, GFI, AGFI, RMSEA, CFI, and PCFI. Bagozzi and Yi (1988) suggested that a smaller ratio of χ2 to its degrees of freedom is better while the revised ratio of this study was 4.10. Hair et al. (1998) claimed that the GFI value and the AGFI value being closer to 1 are better while the corrected GFI and AGFI for this study were 0.86 and 0.83, respectively. Browne and Cudeck (1993) argued that the best RMSEA value should be less than 0.08, and the RMSEA value in this study was corrected to 0.07. The CFI standard value should be greater than 0.90, and the CFI value was corrected to 0.93. The PCFI value must be at least greater than 0.50, and the revised PCFI was 0.81. These findings showed that the overall fit indices of the research results reached the criteria, as seen in Table 7.

**Table 7:** Overall model fit analysis.

Goodness of fit index	Allowable range	Revised model	Model fit determination
χ^2^ (Chi-square)	The smaller, the better	980.91	
Ratio of χ^2^ to the degree of freedom	<3	4.10	Acceptable
GFI	>0.80	0.86	Pass
AGFI	>0.80	0.83	Pass
RMSEA	<0.08	0.07	Pass
CFI	>0.90	0.93	Pass
PCFI	>0.50	0.81	Pass

**Figure 2:**
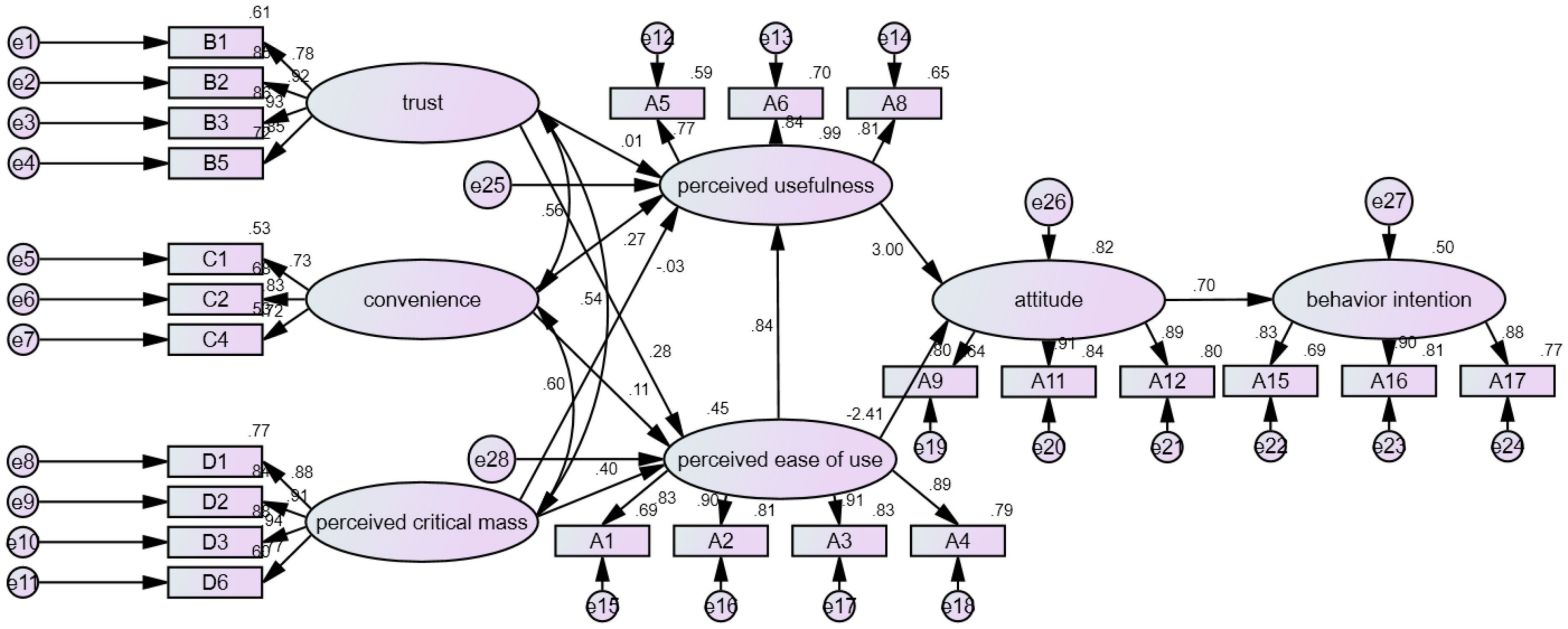
Research model of the impact of online teaching in behavior intention for college students.

In the results of this study shown in Figure 2 and Table 8. H1 is not supported, which means that there is no significant impact of trust on perceived usefulness. This research result is different from those of Sari et al. (2021). This is possibly because during students’ use of the online teaching platform, although most schools adopt the same online teaching platform (such as Microsoft Teams), students think that the learning effect of the online teaching platform is worse than expected due to the influence of software and hardware equipment. H2 is supported, which means that there is significant impact of trust on perceived ease of use. This research result is similar to the findings of Harrigan et al. (2021). The possible reason is that it is more convenient and easier for students to do homework and have online discussions through online teaching platforms than through traditional classroom teaching. H3 is supported, which means that there is significant impact of convenience on perceived usefulness. This research result is similar to that of Hsu (2017). The possible reason is that students can join online courses at a certain time at home, which makes students feel that they can save commuting time, or students can have a different learning experience from the online learning interaction. H4 is not supported, which means that there is no significant impact of convenience on perceived ease of use. This research result is different from the research conclusion. The possible reason is that students can choose their class time and place more freely through online teaching platforms, but they may need to deal with more online procedures (e.g., opening the camera or the microphone), which may not really save their efforts. H5 is not supported, which means that there is no significant impact of perceptual critical mass on perceived usefulness. This research result is different from those of Lee et al. (2013). The possible reason is that when most groups adopt an online teaching platform, relevant personnel of other educational fields will be more willing to adopt this platform, but a lack of choice may affect the students’ learning attitude. H6 is supported, which means that there is significant impact of perceived critical mass on perceived ease of use. This research result is similar to that of Lee et al. (2013). The possible reason is that students will be more confident in the effective learning brought by the online teaching system when the system that they are using is used by more people. H7 is supported, which means that there is significant impact of perceived ease of use on perceived usefulness. This research result is consistent with that of Byun et al. (2018). The possible reason is that students will be more aware of the learning benefits brought by the online teaching platform when they think that the platform is easy to operate. H8 is supported, which means that there is significant impact of perceived usefulness on attitude. This research result is s consistent with that of Islami et al. (2021). The possible reason is that students will have a more positive attitude toward the online teaching platform when they are more aware of the learning benefits brought by the platform. H9 is supported, which means that there is significant impact of perceived ease of use on attitude. This research result is similar to the findings of Chen et al. (2014). The possible reason is that students will have a more positive attitude toward the online teaching platform when they save more time by using the platform. H10 is supported, which means that there is significant impact of attitude on behavior intention. This research result is consistent with that of Chang (2021). The possible reason is that students agree with the benefits gained from the online teaching platform, thus, they are more accepting of the teaching mode of the online teaching platform.

**Table 8:** Empirical results of research hypotheses.

Hypothesis	Path relationship	Path value	Hypothesis established or not
1	There is significant impact of trust on perceived usefulness	0.01	Not established
2	There is significant impact of trust on the perceived ease of use	0.28^*^	Established
3	There is significant impact of convenience on perceived usefulness	0.27^*^	Established
4	There is significant impact of convenience on perceived ease of use	0.11	Not established
5	There is significant impact of perceived critical mass on perceived usefulness	−0.03	Not established
6	There is significant impact of perceived critical mass on perceived ease of use	0.40^*^	Established
7	There is significant impact of perceived ease of use on perceived usefulness	0.84^*^	Established
8	There is significant impact of perceived usefulness on attitude	3.00^*^	Established
9	There is significant impact of perceived ease of use on attitude	−2.41^*^	Established
10	There is significant impact of attitude on behavior intention	0.70^*^	Established

* *p* < 0.05.

## Conclusion and suggestions

### Conclusion

This paper draws the following main conclusions through empirical analysis:

H1 is not supported, which means that there is no significant impact of trust on perceived usefulness.
H2 is supported, which means that there is significant impact of trust on perceived ease of use.
H3 is supported, which means that there is significant impact of convenience on perceived usefulness.
H4 is not supported, which means that there is no significant impact of convenience on cognitive usability.
H5 is not supported, which means that there is no significant impact of perceptual critical mass on the perceived usefulness.
H6 is supported, which means that there is significant impact of perceptual critical mass on perceived ease of use.
H7 is supported, which means that there is significant impact of perceived ease of use on perceived usefulness.
H8 is supported, which means that there is significant impact of perceived usefulness on attitude.
H9 is supported, which means that there is significant impact of perceived ease of use on attitude.
H10 is supported, which means that there is significant impact of attitude on behavior intention.

### Suggestions

Based on the above research results, this paper puts forward some suggestions for reference:

#### For students

It can be seen from the results of this paper that trust has significant impact on perceived ease of use. Therefore, it is suggested that students should first understand the operational interface of the online teaching platform before using the platform. In particular, there are different operational characteristics in different online teaching platforms, and the products with a certain quality can make consumers generate a sense of trust. As a result, if students can become familiar with the operational interface in advance, it is easier for them to find the course and join the video conference, if the login screen of a laptop is different from that of a phone, prior knowledge can help solve this problem in a timely manner, and students can more easily use the online teaching platform when they have prior knowledge of platform use. However, the results also show that perceived critical mass has significant impact on perceived ease of use, thus, students are suggested to ask seed teachers of the platform for help using the system, as well as the steps for learning in the early stages before teaching. Such prior knowledge helps students to understand the course content in advance, which facilitates discussions with teachers and peers through the online chat room during formal teaching, and reduces the challenges faced by students in remote teaching. In other words, the more successful role that critical mass plays, the more students will recognize the learning effect and smooth operation brought by the online teaching system. In addition, the results of this paper show that attitude has significant impact on behavior intention. Consequently, students are suggested to build an environment where they can focus when using the online teaching system, meaning they should avoid excessively noisy spaces and cultivate their spirit of independent learning by concentrating on learning. At the same time, when learning is supplemented by teachers’ diversified teaching materials to stimulate interest-oriented learning, and learning is not completely limited by the curriculum structure, it means students can achieve their learning goals faster through an online teaching system, and have a positive attitude toward completing the learning activities more easily and efficiently, which makes students more willing to adopt the online teaching system.

#### For teachers and school-related units

The results of this paper demonstrate that perceived ease of use has significant impact on perceived usefulness. Therefore, school-related units are suggested to adopt online teaching software or interfaces suitable for teachers and students, avoid messy interface designs, complex system operations, and unfriendly interface designs, and consider the compatibility between mobile phones and different operating systems. In particular, more attention should be paid to the technical support services of traffic and troubleshooting on the online teaching platform. With all these strategies, students will feel the online teaching platform is easy to use, and then, recognize the learning experience and teaching results brought by it. In addition, the results show that perceived usefulness and perceived ease of use have significant impacts on attitude. Therefore, teachers are suggested to make good use of the functions and advantages of the online teaching platform; for example, teachers can increase the flexibility of teaching by making use of media technology, and remote teaching can present diversified teaching styles to further improve students’ learning motivation and learning responsibility through interactions on various media (such as the Kahoot app, internet, and video streaming). In addition, an online teaching platform can help enhance students’ willingness to learn through interactions and receiving real-time feedback during class; for example, students’ class assignments through traditional teaching methods can only be read by teachers, while the online teaching platform allows teachers and students to exchange ideas and learn from the questions raised by other students during class, which can improve both teaching and learning. When students are satisfied with the teaching content and interactive process provided by the online teaching platform, they will be more willing to use the platform. In addition, the results show that convenience has significant impact on perceived usefulness; therefore, teachers are suggested to help students understand the operation of the platform interface through many methods, such as recording an operational video and providing it to students to watch repeatedly. All these suggestions can further reduce students’ troubles caused by the unfamiliar operations and enhance their recognition of the learning effect of the online teaching platform.

#### For future research

It can be seen from the results that three research hypotheses in this paper are not supported. In particular, the hypotheses regarding trust and convenience for perceived usefulness are not supported. It is suggested that in future research, the cognition and usage behavior of college students regarding online teaching software or platforms can be further explored; for example, exploring the important aspects and cognitive aspects of students for an online teaching platform can be conducted through analysis of the important attributes, or exploring the actual process of students’ use of online teaching software through in-depth interviews, on-site observations, and other diversified methods, and recording the problems that students often meet in the process of online teaching, which can be provided to students, in order that questionnaires for future relevant research can be improved. In addition, some specific situations can be considered by future research; for example, studies should explore the possible gaps between teachers and students during digital learning, as caused by the urgent use of online teaching under the pandemic, as well as other factors (such as the digital divide, the quality of audio and video, the content of digital textbooks, and the suitability of using online teaching for specific subjects) that may affect students’ perception of the usefulness of online learning platforms. Future research results are expected to be enriched through the above-mentioned related strategies.

## Data availability statement

The original contributions presented in the study are included in the article/supplementary material, further inquiries can be directed to the corresponding author.

## Author contributions

All authors listed have made a substantial, direct, and intellectual contribution to the work and approved it for publication.

## Conflict of interest

The authors declare that the research was conducted in the absence of any commercial or financial relationships that could be construed as a potential conflict of interest.

## Publisher’s note

All claims expressed in this article are solely those of the authors and do not necessarily represent those of their affiliated organizations, or those of the publisher, the editors and the reviewers. Any product that may be evaluated in this article, or claim that may be made by its manufacturer, is not guaranteed or endorsed by the publisher.
